# Supplementary material for: Inferring TF activities and activity regulators from gene expression data with constraints from TF perturbation data
Source: Bioinformatics. 2020 Dec 22;37(9):1234–45. doi: 10.1093/bioinformatics/btaa947 (PMC8189679; doi:10.1093/bioinformatics/btaa947)
Supplement: btaa947_Supplementary_Data [file btaa947_supplementary_data.zip › Ma&Brent2020Supplement2ndSubmission.pdf]

SUPPLEMENTARY FILES

S1_50-TF_ChIP-CC_binary_network_with_signs_from_TFKO.csv
S2_50-TF_ChIP-CC_binary_network_with_signs_from_ZEV.csv
Signed binary networks of edges between TFs and target genes. Edges come from top scores in ChIP dataset (1), while signs come from the correlation between TF-mRNA and target gene mRNA in either the TFKO (2) or the ZEV (3) dataset. See Network construction below for more details.

S3_50-TF_ChIP-CC_network_with_optimized_values_from_TFKO.csv
S4_50-TF_ChIP-CC_network_with_optimized_values_from_ZEV.csv
Optimized control strength values for edges between TFs and target genes. Edges and signs come from the binary networks (Files S1 and S2), while the quantitative values come from fitting to gene expression profiles of either the TFKO or the ZEV dataset.

S5_50-TF_TFA_values_optimized_with_ChIP-CC-TFKO_on_ZEV.csv
Optimized TFA values on ZEV dataset, using the ChIP-CC values that were optimized on TFKO.

S6_50-TF_TFA_values_optimized_with_ChIP-CC-ZEV_on_TFKO.csv
Optimized TFA values on TFKO dataset, using the ChIP-CC values that were optimized on ZEV.

S7_50-TF_ChIP-PC_binary_network_with_signs_from_TFKO.csv
S8_50-TF_ChIP-PC_binary_network_with_signs_from_ZEV.csv
S9_50-TF_ChIP-PC_network_with_optimized_values_from_TFKO.csv
S10_50-TF_ChIP-PC_network_with_optimized_values_from_ZEV.csv
S11_50-TF_TFA_values_optimized_with_ChIP-PC-TFKO_on_ZEV.csv
S12_50-TF_TFA_values_optimized_with_ChIP-PC-ZEV_on_TFKO.csv
See description for files S1-S6. Signs for the binary networks come from the gene expression response of targets to the perturbation of TFs in either the TFKO or the ZEV dataset. Specifically, if a target gene decreases or increases expression between a TF deletion sample and the WT, the edge between the TF and target gene is positive or negative respectively. If a target gene decreases or increases expression between a TF induced sample and the WT, the edge between the TF and target gene is negative or positive respectively.

S13_50-TF_DE-PC_binary_network_with_signs_from_TFKO.csv
S14_50-TF_DE-PC_binary_network_with_signs_from_ZEV.csv
S15_50-TF_DE-PC_network_with_optimized_values_from_TFKO.csv

S16_50-TF_DE-PC_network_with_optimized_values_from_ZEV.csv
S17_50-TF_TFA_values_optimized_with_DE-PC-TFKO_on_ZEV.csv
S18_50-TF_TFA_values_optimized_with_DE-PC-ZEV_on_TFKO.csv
See description for files S7-S12. Signs and edges for the binary networks come from the gene expression response of targets to the perturbation of TFs in either the TFKO or the ZEV dataset.

S19_50-TF_PWM-PC_binary_network_with_signs_from_TFKO.csv
S20_50-TF_PWM-PC_binary_network_with_signs_from_ZEV.csv
S21_50-TF_PWM-PC_network_with_optimized_values_from_TFKO.csv
S22_50-TF_PWM-PC_network_with_optimized_values_from_ZEV.csv
S23_50-TF_TFA_values_optimized_with_PWM-PC-TFKO_on_ZEV.csv
S24_50-TF_TFA_values_optimized_with_PWM-PC-ZEV_on_TFKO.csv
See description for files S7-S12. Edges for the binary networks come from top scores of motif scanning, and signs come from the gene expression response of targets to the perturbation of TFs in either the TFKO or the ZEV dataset.

S25_94-TF_Union-PC_binary_network_with_signs_from_TFKO_and_ZEV.csv
S26_94-TF_Union-PC_network_with_optimized_values_from_TFKO_and_ZEV.csv
S27_94-TF_TFA_values_optimized_with_Union-PC-TFKO-ZEV_on_2.0p_glucose.csv
S28_94-TF_TFA_values_optimized_with_Union-PC-TFKO-ZEV_on_0.02-0.2p_glucose.csv
S29_94-TF_TFA_values_optimized_with_Union-PC-TFKO-ZEV_on_Regulators.csv
S30_94-TF_TFA_values_optimized_with_Union-PC-TFKO-ZEV_on_Zaman.csv
See description for files S7-S18. Signs and edges for the binary network comes from non-conflicting edges of ChIP-PC from TFKO, ChIP-PC from ZEV, DE-PC from TFKO, and DE-PC from ZEV. Network edges are optimized on both TFKO and ZEV. TFA values used CS values optimized on TFKO and ZEV to fit glucose influx data, all TFKO to include samples beyond just TF deletion strains, and a dataset used in the paper to further analyze TF regulation (4).

S31_Literature_curated_regulators_of_TFA.csv
A curated map of TFA regulators. The first two columns are the systematic and common names of TFs. The next two columns are the systematic and common names of the TFA regulators. The fifth column indicates the direction of effect from the regulation, with -1 if the regulator decreases the activity of the TF, 1 if the regulator increases the activity of the TF, and 0 if the direction is unknown or not straightforward, i.e. part of a feedback loop. The next column is a brief description of the regulatory mechanism, and the last column records the PMID of the paper that describes the regulatory relationship.

EVIDENCE SUPPORTING INFERRED TFA REGULATORS

The inferred TFAs for potential TF activity regulators, obtained by analyzing expression data from refs. are provided as File S29 and S30. We manually curated the results to obtain the TFA regulatory interactions shown in Figure 5. The evidence supporting most of the hypothesized novel TF regulatory interactions is shown in Figure 5B, C.

Some of the findings shown in Figure 5A are as follows. We confirmed that Gcn2 activates Gcn4 (5-8), Gcn1 activates Gcn4 (probably via its effects on Gcn2 (9)), and Ure2 represses Gln3 (probably by anchoring it to the plasma membrane, (6, 7)). We also saw evidence that Ure2 may activate Gcr2 directly or indirectly. We confirmed the well-known role of TORC1 as a repressor of Gln3 and Gcn4 activity (10-12) but did not see unequivocal evidence that it represses Msn2 as previously reported (11, 12). Our analyses showed that Grr1 represses Gln3, probably via Npr2 (13), which our analysis confirms as an activator of Gln3 (6)), and activates Gcr2 (probably indirectly; Fig. 5B). Although it has not been previously reported that Grr1 activates Gcr2, Grr1 is known to be required for glucose suppression, so activation of glycolytic genes via Gcr2 would be a consistent role. We also found that Grr1 represses Gcn4 and Msn2 (Fig. 5B), consistent with its being active in nutrient-replete conditions. Since Grr1 activates Gcr2 and represses the other three, a transient spike in its activity upon glucose influx could explain the upward and downward spikes we see in the activities of the four TFs. We confirmed that the SWI/SNF chromatin remodeling complex contributes to the activities of Gln3 (14, 15) and Gcn4 (8) and discovered that it also works with Gcr2. We confirmed that PKA represses Msn2 (6, 7, 11, 12, 16) and saw evidence that it also represses Gcr2 (Fig. 5C), which has not been previously reported. We confirmed that Snf1 represses Gcn4 in the absence of glucose and amino acids and saw evidence that it weakly represses Gln3 (Fig. 5B, D), contrary to previous claims that Snf1 activates Gln3 (12, 17). In summary, we have identified several likely regulators of Gcr2 activity, about which little was previously known, and discovered that Grr1, previously known for its role in glucose repression, is probably a positive regulator of glycolysis (via Gcr2) and a negative regulator of stress-induced TFs. These novel observations, which were mined from gene expression data via TF activity inference, constitute a rich trove of hypotheses for future experimental investigation.

METHODS

<u>Datasets Used</u>

Yeast TFKO data The microarray expression data of 1,484 single gene knockout strains (2) was downloaded from

http://deleteome.holstegelab.nl/data/downloads/deleteome_all_mutants_controls.txt A sample using expression level of 0 for all genes was assumed in order to stand in for WT.

Yeast ZEV induction data The microarray expression data of 199 single gene ZEV induction strains (3) was downloaded from https://storage.googleapis.com/calico-website-pin-public-bucket/datasets/pin_tall_expression_data.zip Only the column labeled log2_cleaned_ratio was considered for this work. A sample using expression level of 0 for all genes was assumed in order to stand in for the 0min timepoint, when induction of over-expression had not yet started.

Yeast ChIP-chip data P-values that represent TF binding significance from ChIP-chip experiments (1) were downloaded from http://younglab.wi.mit.edu/regulatory_code/GWLD.html Values were transformed to negative log10 p-values for the purpose of treating them as confidence scores, where greater values indicate greater support.

Yeast PWM data Position weight matrices for S. cerevisiae motifs in the ScerTF database (18) were downloaded from http://stormo.wustl.edu/ScerTF/ Values from FIMO scanning (19) for motif hits were transformed to negative log10 p-values for the purpose of treating them as confidence scores, where greater values indicate greater support. If multiple hits were found between linking the same TF and target gene, the maximum score was used.

Yeast double-deletions data The microarray expression dataset of 69 double-deletion strains (20) was downloaded from http://www.holstegelab.nl/publications/GSTF_geneticinteractions/ A sample using expression level of 0 for all genes was assumed in order to stand in for WT.

Yeast time course data
The microarray expression data of 9 time-points (0min, 3, 7.5, 15, 30, 60, 110, 150, 300min) after 2% glucose influx for WT strains (21) was downloaded from http://www.holstegelab.nl/publications/glucose_regulatory_system/ A sample using expression level of 0 for all genes was assumed in order to stand in for 0min.

The microarray expression data of 13 (0min, 2, 4, 6, 8, 10, 15, 20, 30, 45, 90, 120, 150) and 15 (0min, 3, 5, 7, 10, 15, 20, 30, 45, 90, 120, 150, 180, 210, 240) time-points after 0.02% and 0.2% glucose influx for WT strains (22) was downloaded from GEO with accession ID GSE4158. A sample using expression level of 0 for all genes was assumed in order to stand in for 0min.

The microarray expression datasets of 5 time-points (0min, 20, 40, 60, 80) for multiple conditions and multiple strains (4) were downloaded from https://puma.princeton.edu/cgi-bin/publication/viewPublication.pl?pub_no=524. Where possible, all samples were re-scaled to use the WT in 3% glycerol condition as the reference, and a sample using expression level of 0 for all genes was assumed in order to stand in for this reference.

Network construction

The actual networks constructed by the methods below are all provided as supplementary files. Code for constructing networks from ranked lists of edges is available at https://doi.org/10.5281/zenodo.4050573 Construction of the ChIP network (Files S1-2, S7-8) is illustrated in Figure 1B and described in the figure legend. Our general network construction algorithm is described in detail below.

First, all possible TF-target interactions are first ranked according to the strength of evidence that the TF regulates the potential target gene. We did this for yeast (*S. cerevisiae*), ranking edges according to their negative log p-value in a comprehensive ChIP-chip dataset, their absolute differential expression in a TF perturbation sample, or their maximum negative log p-value in a comprehensive PWM dataset. To integrate data sources, the edges can be rank-averaged at this point, though the performance of such integrated networks is not shown in this paper.

To build a network map, we first dropped all but the top 1,250 edges. Then, starting from the top, edges were added until 50 TFs were included. If there were not enough TFs, the total number of edges considered was iteratively increased by 25 until at least 50 TFs could be recovered. Any remaining edges were only included if they emanated from TFs already in the map. This initial map was then checked for any TFs with a single target gene, and any set of TFs with identical target genes. These TFs and their target genes were removed from the map. Single-target TFs were removed to avoid having TFA values dependent on only one feature, which would be extremely vulnerable to noise or measurement error. TFs with identical targets were removed because it is impossible to separate the contributions to gene expression (i.e. the control strength matrix is not of full rank). If necessary, we returned to the list and added edges that were previously skipped over, repeating all steps until the network holds steady at 50 TFs.

The ChIP network (Files S1-2, S7-8) considered the top 1,250 edges, using 1,104 of them to build a network of 50 TFs and 778 genes. The minimum score (negative log adjusted p-value) was 4.37.

For the differential expression (DE) network based on TFKO (File S13), the top 1250 edges were not sufficient to build a network of 50 TFs when using the TFKO dataset, so additional edges were considered in increments of 25 until the top 1400 edges was found to be sufficient. 1,283 of them were used to build a network of 50 TFs and 573 genes. The minimum score (absolute logFC of gene expression) was 1.25.

The DE network based on ZEV (File S14) considered the top 1,250 edges, using 890 of them to build a network of 50 TFs and 686 genes. The minimum score (absolute logFC of gene expression) was 1.67.

The PWM network (Files S19-20) considered the top 1,250 edges, using 1,023 of them to build a network of 50 TFs and 894 genes. The minimum score (maximum negative log p-value) was 9.19.

To add correlation-based sign constraints (eg. ChIP-CC, Files S1-2), the direction of correlation between the TF and target gene's expression levels across samples was calculated. Samples where a network TF was directly perturbed were not included in the correlation calculation to ensure that these constraints were based on general correlation trends and not based on gene expression response to direct perturbation of an assigned TF regulator.

To add perturbation-based sign constraints (eg. ChIP-PC, Files S7-8), the direction of a gene's expression in the perturbation sample of its TF was used. For TFKO-based constraints, the sign was reversed to indicate that the TF likely activates a gene that decreases expression in its absence, and vice versa. For ZEV-based constraints, the sign was used directly.

For networks using lower quality edges, the goal was to build networks where the support for all edges decreased, without overlap. In anticipation of situations like the DE network based on ZEV, where the total number of edges had to be increased beyond 1250 in order to obtain a network of 50 TFs, a generous 2,000 edges were selected to comprise a "block," even though the networks themselves never needed all 2,000 edges. Block 1 networks are the same networks described above, while the Block 2 networks were created after reassigning ranks when the top 2,000 edges were zero-ed out, the Block 4 networks were created after reassigning ranks when the top 6,000 edges were zero-ed out, etc... The number of edges considered to build each network was kept to 1,250 whenever possible, with the only exceptions being two DE networks based on TFKO, where Block 1 used 1,400 as described above, and Block 2 used 1,450 edges.

The ChIP and DE extended networks were built for direct comparison with combining the TFA values inferred from ChIP-PC and DE-PC. The union of ChIP-PC and DE-PC based on TFKO was 77 TFs, and the union of ChIP-PC and DE-PC based on ZEV was 80 TFs. Therefore, the ChIP and DE extended networks needed to cover the same number of TFs, depending on which dataset was being used for CS optimization instead of TFA validation. The number of edges considered was extended proportionally; eg. a network increase from 50 to 77 TFs started with an increase from 1,250 to 1250*(77/50) =1,925 edges.

The ChIP extended network for optimizing on TFKO considered the top 1,925 edges, using 1,866 of them to build a network of 77 TFs and 1,196 genes. The minimum score (negative log adjusted p-value) was 3.70.

The ChIP extended network for optimizing on ZEV considered the top 2,000 edges, using 1,968 of them to build a network of 80 TFs and 1,250 genes. The minimum score (negative log adjusted p-value) was 3.64.

The DE extended network for optimizing on TFKO considered the top 3,575 edges, using 3,284 of them to build a network of 77 TFs and 1,213 genes. The minimum score (absolute logFC of gene expression) was 0.85.

The DE extended network for optimizing on ZEV considered the top 2,000 edges, using 1,707 of them to build a network of 80 TFs and 1,019 genes. The minimum score (absolute logFC of gene expression) was 1.45.

The Union-PC network (File S24) was built from the union of ChIP-PC edges, and edges from the two DE-PC networks, one derived from the TFKO data and the other from the ZEV. This started out as a set of 3,133 unique edges between 96 TFs and 1592 target genes. Among the edges in the ChIP network, 413 have conflicting sign constraints between the ZEV and TFKO datasets and were therefore discarded. Among the 42 edges in both the ZEV- and TFKO-based DE networks, none have conflicting sign constraints, so all edges were kept. Finally, among the 90 edges found in both the trimmed ChIP network and DE networks, none had conflicting sign constraints, which left us with 2,732 edges. After filtering out two TFs that were left with only a single target gene due to the loss of ChIP edges, we were left with 94 TFs, 1,416 target genes, and 2,731 edges.

<u>Model-Fitting</u>

Given a network of edges between TFs and genes, random values between -10 and 10 are generated to stand in for the control strengths of those edges, as well as the baseline expression level of genes. Any sign constraints on control strength parameters are then checked, flipping the signs of the random control strength values as necessary. For this paper, this process was repeated to create 20 random starts for each network structure.

Each random start is used to create a linear optimization problem, where activity values are optimizable, non-negative parameters to minimize the squared error between measured gene expression and model predicted gene expression. For this paper, the gene expression set used in the initial fitting was composed of one perturbation sample for each of the network TFs and a WT sample. The activity of TFs deleted in a sample are set to zero, while the activity of TFs over-expressed in a sample are constrained to be greater than its activity in wild-type samples. In this model, multiplying a TF's activity values by a scaling factor and dividing its control strengths by the same factor leaves the predicted expression levels unchanged. Since the scale cannot be determined from the expression data, we constrain the mean activity of each TF, across all samples, to be one. After optimization, the random control strength values are discarded, and a new linear optimization problem is created, where control strengths and baseline values are optimizable parameters to minimize the squared prediction error. The control strengths of TFs are constrained to be negative or positive if the edge is known to be activating or repressive, respectively. This back-and-forth process, known as an iteration of bi-linear optimization, is repeated until the variance explained from fitting the new control strength values on the second dataset peaks, or until a maximum of 100 iterations is reached.

Finally, across the random starts, the set of parameters that achieved the greatest variance explained on the optimization set is selected for evaluation.

When optimizing for new baseline and activity parameters using an existing set of control strength parameters, the bi-linear framework is maintained. New activity parameters are optimized first, and the mean activity of each TF across samples is not constrained to one. After optimizing for activity, new baseline parameters are optimized. This iterates until the improvement in variance explained falls below 1%. For this paper, the gene expression set for this second fitting consisted of 179 perturbation samples and a WT sample.

When optimizing the Union-PC network on both the ZEV and TFKO datasets, the bi-linear approach remains the same, where TFA values for all samples are optimized against 20 sets of random CS values, before new CS values are optimized against the

optimized TFA values. However, due to the datasets coming from different conditions, labs, and microarray technology, different baselines were optimized for the different datasets to compensate for any constant shifts in gene expression measurements. One perturbation sample for each TF in the network from each of the two datasets was included in the gene expression set, plus a WT sample for each of the two datasets. The set of TFA and CS values that achieved the greatest variance explained on the combined ZEV and TFKO dataset was selected for evaluation.

All optimization in this paper was done using Gurobi, an optimizer that offers free Academic licenses (23). Code for this optimization is available at https://doi.org/10.5281/zenodo.4050573

Calculating Evaluation Metrics

To ensure the inferred TFA values being evaluated is entirely blind of the criteria being evaluated, optimization of model parameters is done in two steps. The initial step optimizes all parameters to fit a dataset, either TFKO or ZEV. During this optimization, constraints can be applied to the activity parameters based on the known perturbations in the optimization dataset, as well as to the control strength parameters based on the known DE of target genes.

For the second step, optimized control strength parameters are used to optimize new baseline and activity parameters for a separate dataset. This second optimization does not allow activity parameters to be constrained based on known perturbations in the new dataset, nor for the control strength parameters to be updated based on conflicting DE of target genes.

The first three metrics explained below can be evaluated using code available here: https://doi.org/10.5281/zenodo.4050573

Direction of Perturbation To predict the direction of TF perturbation in a given sample, we compare the TF's activity in that sample to its activity in the WT sample. The percent of samples correctly predicted is calculated for where one of the two datasets serves training data and the other as the test data. We then swap the roles, with the dataset that was previously serving as training now serving as test, and vice-versa. The final score as an average between the two. Below, we refer to this averaging procedure as the average of the two train-test directions. To calculate the p-value, a binomial test is calculated for a 50% random chance of guessing the correct direction, where the number of trials is the total number of TFs evaluated in each dataset, averaged using Fisher's combined probability test.

Median Rank Percentile To predict the perturbed TF, we log and standardize all activities to Z-scores within each TF, then rank all standardized log activities in each sample. If a sample involves a TF overexpression, we rank from highest to lowest; if a sample involves a TF knockout or knockdown, we rank from lowest to highest. The rank 1 TF is given the rank percentile of 100%, while subsequent TFs are $\left(100 - \frac{(rank-1)}{numTFs}\right)$ %. The rank percentile of the perturbed TF is used as an accuracy score for each sample, and the median rank percentile is used as an accuracy score for each dataset (TFKO and ZEV), with the final score being an average between the two train-test directions. To calculate the P-value, a binomial test is calculated where the number of trials is the number of samples evaluated, half are successes, and the probability of success is the probability of randomly achieving the final score or better as a rank percentile. This represents the desired null model of how likely it is that half the TFs achieve at least the median rank percentile, given the random probability of that rank percentile.

Positive correlation The fraction of TFs whose measured mRNA level and inferred activity are positively correlated is calculated for 1,000 bootstraps from the 180 samples used in the second fitting, and the median from the bootstrapping for each train-test direction is then averaged to return as the final score. To calculate the P-value, a binomial test is calculated for a 50% random chance of getting a positive correlation, where the number of trials is the total number of TFs evaluated in each dataset, averaged using Fisher's combined probability test. The statistical significance of the individual TFs' correlations is not considered, since the bootstrapping provides robustness against sampling error.

Known regulators of TF activity We compiled a map of TF activity regulators by curating literature on all network TFs, looking for published work that proposed a specific mechanism of activity regulation, such as phosphorylation, nuclear localization, or complex formation (File S31). To evaluate these literatures supported relationships, we rank the change in all standardized activities in samples where a known regulator of TF activity is perturbed compared to the wild-type sample. As the literature does not always give a clear, unique direction to the expected change in TF activity, we rank from highest to lowest absolute value. The rank percentile of the regulated TF is used as an accuracy score for the relationship, and the median rank percentile is used as an accuracy score for the whole dataset.

ZEV time course data The expected behavior of a TF in the yeast ZEV induction dataset is an increasing sigmoid in response to the induced transcription of its gene. To evaluate whether we can recover that expected response pattern, we inferred TFAs for the whole ZEV time course, using a network, constraints, and CS matrix derived from the TFKO data. For each time point, (2.5, 5, 10, 15, 20, 30, 45, 60, and 90 minutes after

induction of TF overexpression, though not every time point was available for every TF), we computed a log fold change of the induced TF's inferred activity, relative to the inferred activity at time 0. We then fit a 4-parameter, sigmoidal, saturating curve to the time series:

$$h_0 + \left(\frac{h_1 - h_0}{1 + e^{\beta(x-t)}}\right)$$

To eliminate curves that fit poorly, we tried several different thresholds on the variance explained by the sigmoidal fit. For each threshold, we calculated the fraction of fits that showed increasing activity throughout the time series (expected behavior) rather than decreasing activity.

Multiple Perturbed TF Identification task For the double deletion set, we want to score our ability to predict the perturbed TFs in any sample where a network TF is deleted, so we first check each sample if the knocked-out TFs are in the network. If none are, we skip the sample, and if one is, we rank and score in the same way we would rank and score a sample with a single TF knock-out. If both TFs are in the network, the standardized log2 activity values are ranked twice, once without including the first TF, and once without including the second TF. The rank percentile of the perturbed TF is used as an accuracy score for each ranking, and the median rank percentile is used as the summary accuracy score.

Calculating CS Condition Independence To evaluate the condition independence of CS values, we wanted to calculate the correlation of CS values inferred in two different growth conditions. The Union-PC network was optimized using only the TFKO data (synthetic complete medium with 2% glucose and no nutrient limitation) and then only the ZEV data (minimal medium with 2% glucose in phosphate-limited, continuous-flow chemostats). Of the 94 TFs in the network, 75 of them had at least 5 targets, which we chose as a minimum since correlations across fewer data points would not be very meaningful. The average correlation for 1,000 bootstrapped samples of the target gene set is calculated for each TF, between the CS values optimized on the two datasets.

Calculating CS correlation with binding data To evaluate whether the control strengths inferred for the targets of a TF would correspond to the strength with which the TF binds those targets, we turned to binding data obtained by the transposon calling cards method (24, 25). In this method, a TF is linked to a transposase, which deposits a transposon in the genome near where the TF is bound. The number of transposons in a gene's promoter is an approximate measure of the amount of time the TF spends bound to that promoter, which we assume to be correlated with true control strength, in most cases. The Union-PC network was optimized on both the TFKO and ZEV data together,

and 11 of the 94 TFs have both Calling Cards data and at least 5 target genes. Using 1,000 bootstrap samplings of target genes, the median correlation between inferred CS and measured transposons was calculated for each TF.

Exploratory Analysis of Inferred TFA values

Glucose influx response pattern After inferring TFA values for three time-courses of glucose influx, we want to summarize the general behavior pattern of TFA response in each. Thus, we plot the inferred activity of each TF as a function of time, fitting both a 4-parameter sigmoid curve and a 6-parameter impulse curve (26), choosing one of the two by the Bayes Information Criterion (27). The sigmoid curve is the same as used for ZEV time-course response pattern analysis, while the impulse curve allows for a return to a new baseline level:

$$\frac{1}{h_1}\left(h_0 + \frac{h_1 - h_0}{1 + e^{\beta(x - t_1)}}\right)\left(h_2 + \frac{h_1 - h_2}{1 + e^{-\beta(x - t_2)}}\right)$$

This results in five general categories of behavior: an upward spike, a downward spike, monotonically increasing, monotonically decreasing, and poor fits (R^2<80%). Using these categories, we could check for expected behavior in response to glucose, as well as consistent behavior across the time-courses.

Response to perturbation of protein complexes The regulatory relationship of complexes and TFs was partially explored using the TFKO dataset, which included knock-out perturbations of many components of complexes, like SWI/SNF and TORC1. In order to analyze the direction and magnitude of effect from perturbing the complex, the median change in standardized log2 activity for a TF across all samples where a component of the complex was knocked-out was compared to other TFs, and to the median change from random sets of the same number of samples.

Supplementary figures

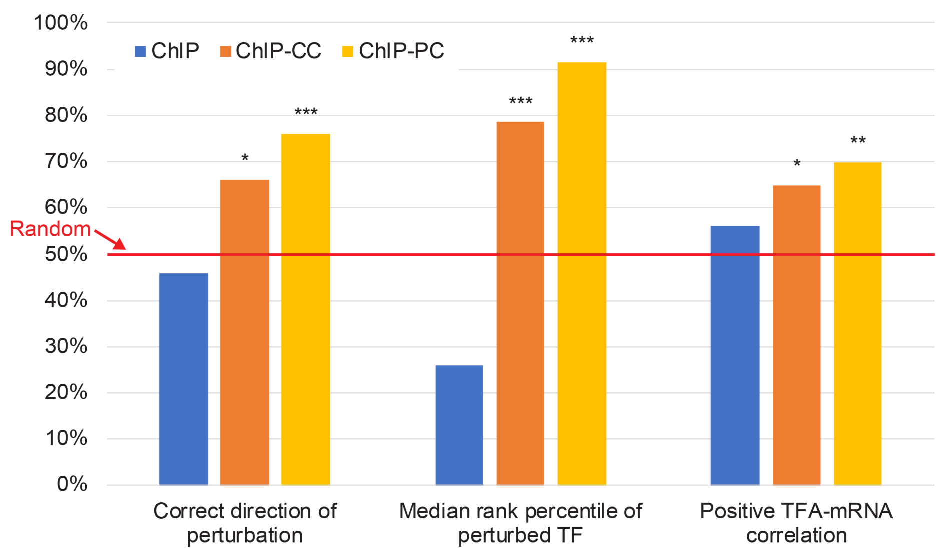

Figure S1
Using ChIP to define the network without constraining the signs (blue) resulted in performance not significantly better than random. Performance of the ChIP network with correlation-based (orange) and perturbation-based (yellow) sign constraints are plotted for comparison.

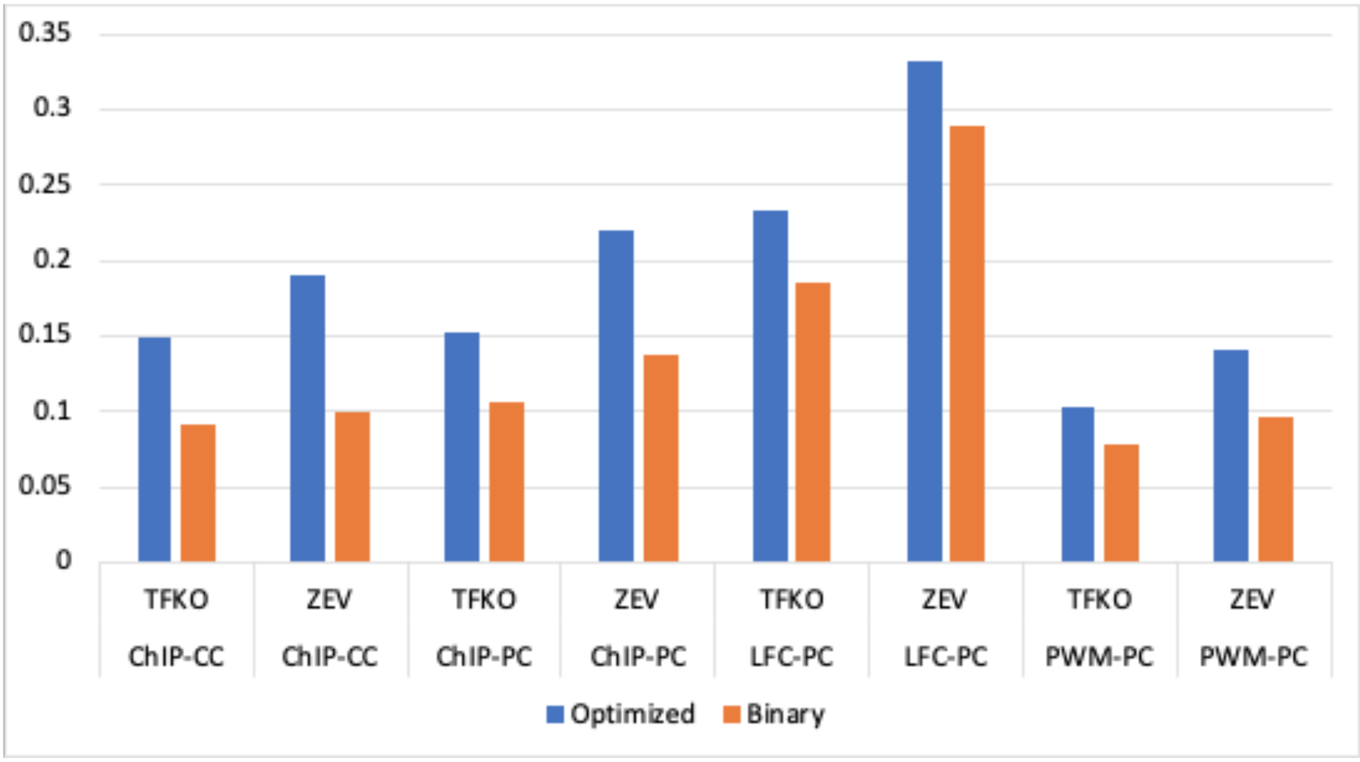

Figure S2
Comparison of variance explained when using CS values optimized on a different data set (blue bars) or signed binary CS values (orange bars). Annotation below each pair of bars indicates the network and constraints used and the data set on which they were optimized. In all cases, both CS matrices are used to infer TFAs and baselines on the other data set and the variance explained is plotted. All pairs of bars show that CS matrices optimized on a different data set yield better fits than signed binary matrices, indicating that optimized CS matrices are, to some degree, transferrable from one growth condition to another.

S3

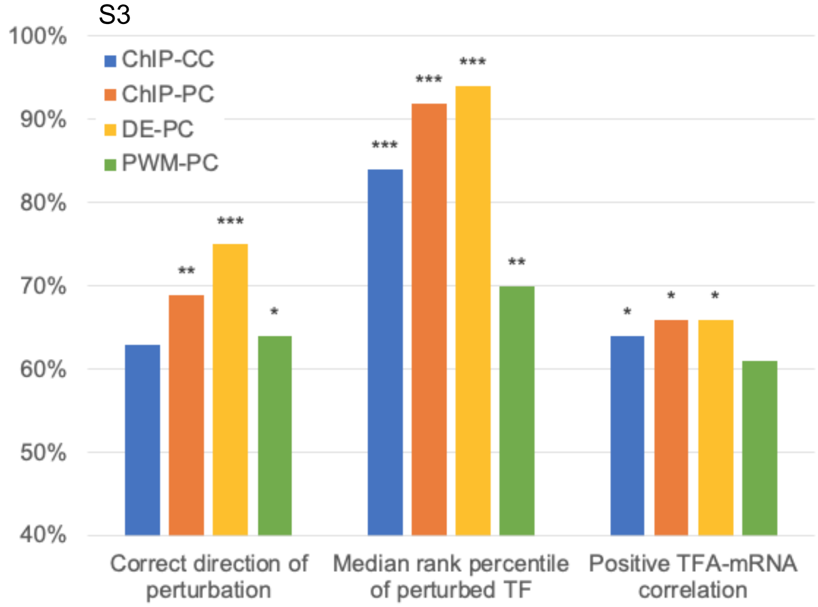

S4

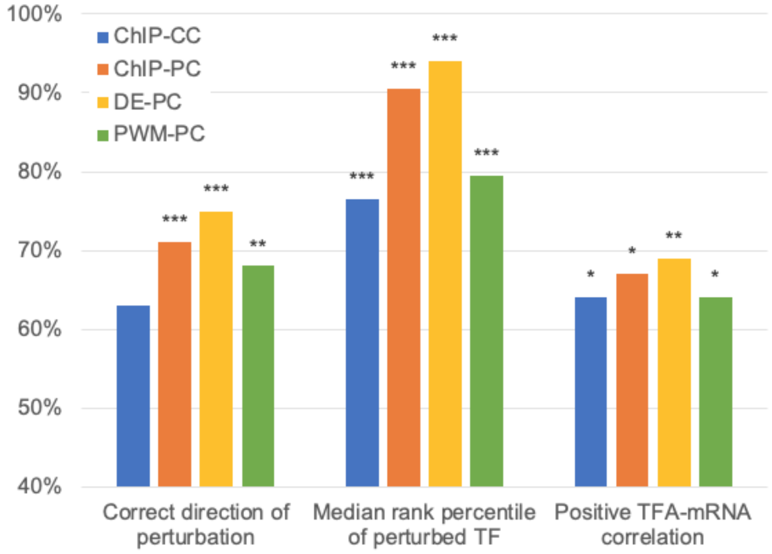

Figures S3 and S4

In comparison with Figure 2A, Fig. S3 and Fig. S4 show the results from using stopping criteria alternative to the peak in variance explained for the second dataset. Fig.S3 shows the results from stopping each random start when the improvement in variance explained from the last iteration drops below 0.1%. This approach shows similar trends between the networks and has the added benefit of not requiring additional data to implement. Fig. S4 shows the results from using the peak in variance explained for held-out samples of the first dataset. Again, we see similar trends in performance. Asterisks above the bars indicate magnitude of significance compared to a random model, with 1, 2, or 3 asterisks representing p-value thresholds of 0.01, 0.001, or 0.0001.

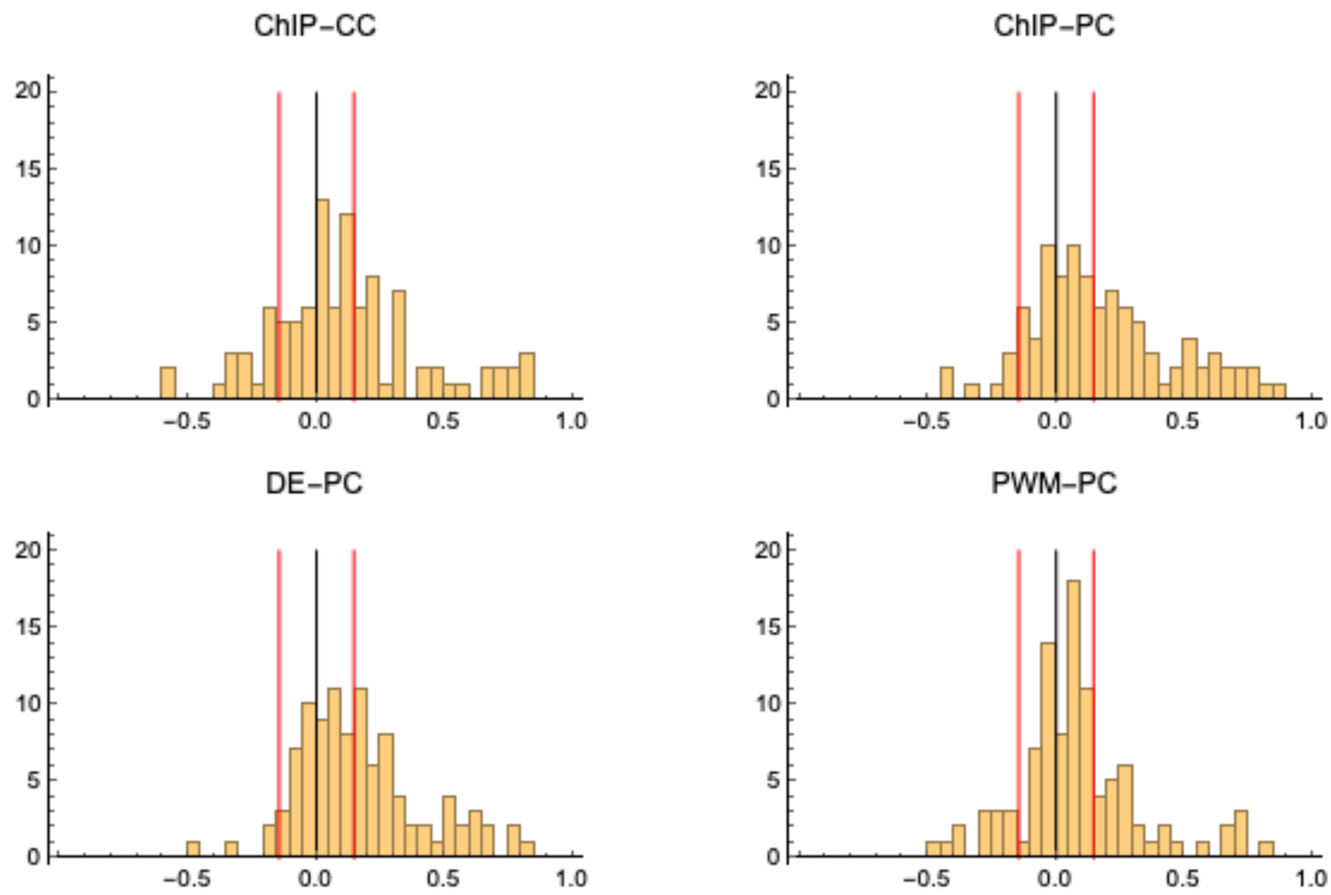

Figure S5
For the *positive correlation* metric, we used bootstrapping to robustly estimate the percentage of TFs with positively correlated TFA and gene expression. This method keeps all 50 TFs in the evaluation. As a more traditional alternative, this figure shows histograms of the correlations calculated between TF activity and gene expression without bootstrapping. Correlations from evaluating both datasets are included, and the red bars at +/- 0.146 indicate where a correlation value for 180 samples passes P<=0.05. ChIP-PC and DE-PC clearly outperform ChIP-CC and PWM-PC, with more significant positive correlations, and fewer significant negative correlations.

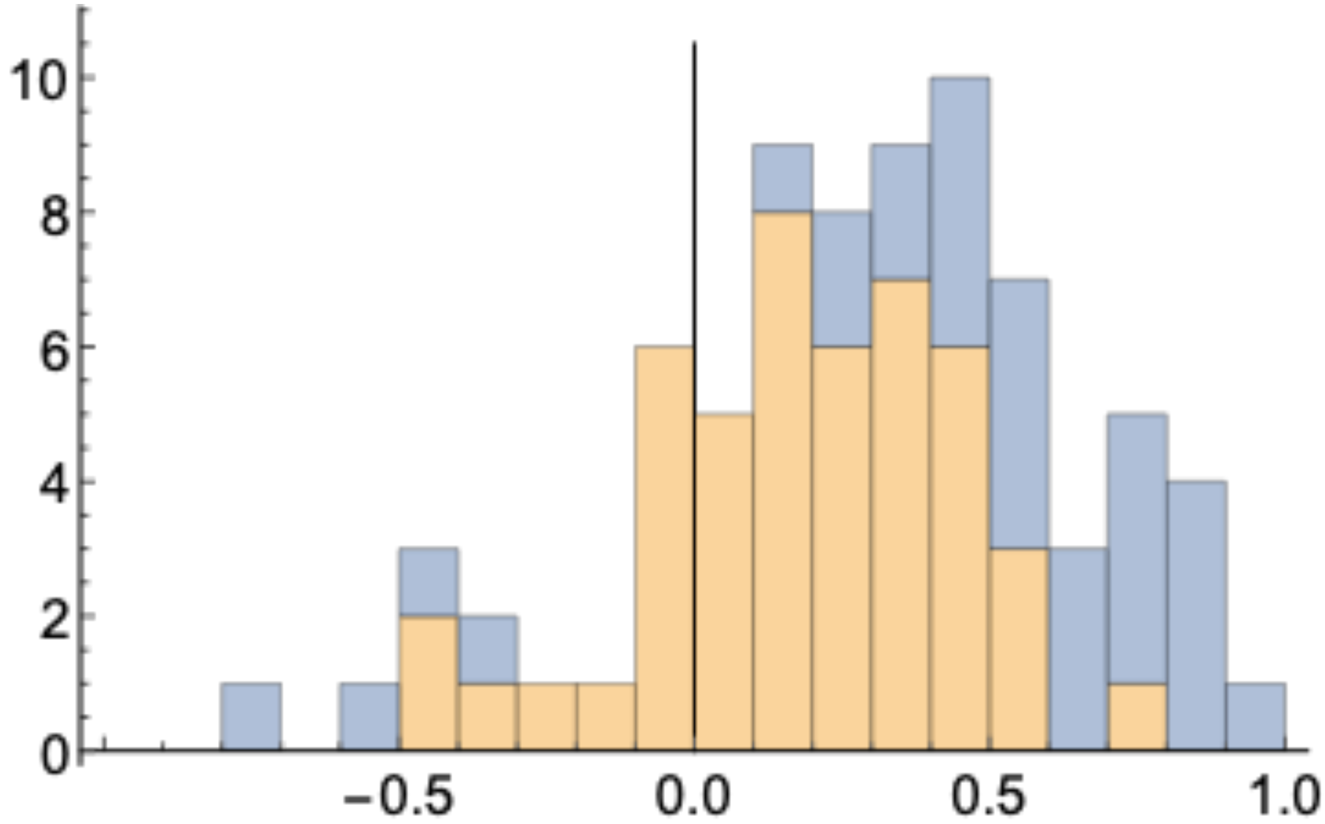

Figure S6
A stacked histogram of correlations between 76 TF's CS values inferred from TFKO data and their CS values inferred from ZEV data, without bootstrapping. Blue indicates counts of TFs with significant correlation at P < 0.05. Most correlations are positive and most significant correlations are also positive.

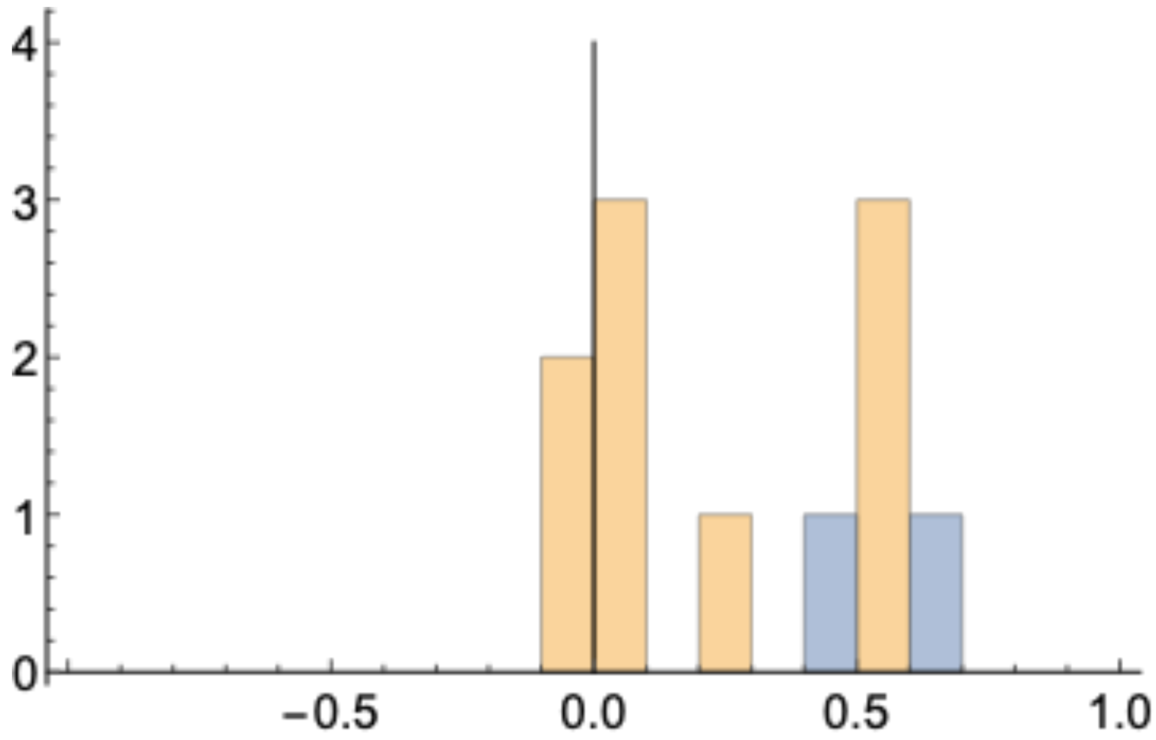

Figure S7
A stacked histogram of correlations between 11 TFs' inferred CS values and Calling Cards binding signal, without bootstrapping. Blue indicates counts of TFs with significant correlation at $P < 0.05$. Calling Cards data is only available for 11 network TFs and correlations are not significant when considered individually because they have only a few targets over which to calculate the correlation. Nonetheless, most correlations are positive, positive correlations tend to have much greater magnitude than negative ones, and the two correlations that are significant when considered individually are strongly positive.

1. C. T. Harbison *et al.*, Transcriptional regulatory code of a eukaryotic genome. *Nature* **431**, 99-104 (2004).
2. P. Kemmeren *et al.*, Large-scale genetic perturbations reveal regulatory networks and an abundance of gene-specific repressors. *Cell* **157**, 740-752 (2014).
3. S. R. Hackett *et al.*, Learning causal networks using inducible transcription factors and transcriptome-wide time series. *Mol. Syst. Biol.* **16**, e9174 (2020).
4. S. Zaman, S. I. Lippman, L. Schneper, N. Slonim, J. R. Broach, Glucose regulates transcription in yeast through a network of signaling pathways. *Mol. Syst. Biol.* **5**, 245 (2009).
5. R. Yang, S. A. Wek, R. C. Wek, Glucose Limitation Induces GCN4 Translation by Activation of Gcn2 Protein Kinase. *Mol Cell Biol* **20**, 2706-2717 (2000).
6. J. R. Broach, Nutritional control of growth and development in yeast. *Genetics* **192**, 73-105 (2012).
7. M. Conrad *et al.*, Nutrient sensing and signaling in the yeast Saccharomyces cerevisiae. *FEMS Microbiol. Rev.* **38**, 254-299 (2014).
8. P. O. Ljungdahl, B. Daignan-Fornier, Regulation of amino acid, nucleotide, and phosphate metabolism in Saccharomyces cerevisiae. *Genetics* **190**, 885-929 (2012).
9. S. Zaman, S. I. Lippman, X. Zhao, J. R. Broach, How Saccharomyces responds to nutrients. *Annu. Rev. Genet.* **42**, 27-81 (2008).
10. K. A. Staschke *et al.*, Integration of general amino acid control and target of rapamycin (TOR) regulatory pathways in nitrogen assimilation in yeast. *J. Biol. Chem.* **285**, 16893-16911 (2010).
11. S. V. Rodkaer, N. J. Faergeman, Glucose- and nitrogen sensing and regulatory mechanisms in Saccharomyces cerevisiae. *FEMS Yeast Res* **14**, 683-696 (2014).
12. C. De Virgilio, The essence of yeast quiescence. *FEMS Microbiol. Rev.* **36**, 306-339 (2012).
13. N. Spielewoy *et al.*, Npr2, yeast homolog of the human tumor suppressor NPRL2, is a target of Grr1 required for adaptation to growth on diverse nitrogen sources. *Eukaryotic cell* **9**, 592-601 (2010).
14. A. Avendano *et al.*, Swi/SNF-GCN5-dependent chromatin remodelling determines induced expression of GDH3, one of the paralogous genes responsible for ammonium assimilation and glutamate biosynthesis in Saccharomyces cerevisiae. *Mol. Microbiol.* **57**, 291-305 (2005).
15. L. Riego, A. Avendano, A. DeLuna, E. Rodriguez, A. Gonzalez, GDH1 expression is regulated by GLN3, GCN4, and HAP4 under respiratory growth. *Biochemical and Biophysical Research Communications* **293**, 79-85 (2002).
16. V. De Wever, W. Reiter, A. Ballarini, G. Ammerer, C. Brocard, A dual role for PP1 in shaping the Msn2-dependent transcriptional response to glucose starvation. *EMBO J.* **24**, 4115-4123 (2005).
17. P. G. Bertram *et al.*, Convergence of TOR-nitrogen and Snf1-glucose signaling pathways onto Gln3. *Mol. Cell. Biol.* **22**, 1246-1252 (2002).
18. A. T. Spivak, G. D. Stormo, ScerTF: a comprehensive database of benchmarked position weight matrices for Saccharomyces species. *Nucleic Acids Res* **40**, D162-168 (2012).
19. C. E. Grant, T. L. Bailey, W. S. Noble, FIMO: scanning for occurrences of a given motif. *Bioinformatics* **27**, 1017-1018 (2011).
20. K. Sameith *et al.*, A high-resolution gene expression atlas of epistasis between gene-specific transcription factors exposes potential mechanisms for genetic interactions. *BMC Biol.* **13**, 112 (2015).

21. E. Apweiler *et al.*, Yeast glucose pathways converge on the transcriptional regulation of trehalose biosynthesis. *BMC Genomics* **13**, 239 (2012).
22. M. Ronen, D. Botstein, Transcriptional response of steady-state yeast cultures to transient perturbations in carbon source. *Proc. Natl. Acad. Sci. U. S. A.* **103**, 389-394 (2006).
23. L. Gurobi Optimization, Gurobi Optimizer Reference Manual. (2020).
24. H. Wang, D. Mayhew, X. Chen, M. Johnston, R. D. Mitra, Calling Cards enable multiplexed identification of the genomic targets of DNA-binding proteins. *Genome Res.* **21**, 748-755 (2011).
25. D. Mayhew, R. D. Mitra, Transposon Calling Cards. *Cold Spring Harb Protoc* **2016**, pdb top077776 (2016).
26. G. Chechik, D. Koller, Timing of gene expression responses to environmental changes. *J. Comput. Biol.* **16**, 279-290 (2009).
27. G. Schwarz, Estimating the Dimension of a Model. *Ann. Statist.* **6**, 461-464 (1978).
